# Effective Harvesting of *Nannochloropsis* Microalgae Using Mushroom Chitosan: A Pilot-Scale Study

**DOI:** 10.3389/fbioe.2020.00771

**Published:** 2020-07-14

**Authors:** Elvis T. Chua, Ajam Y. Shekh, Eladl Eltanahy, Skye R. Thomas-Hall, Peer M. Schenk

**Affiliations:** ^1^Algae Biotechnology Laboratory, School of Agriculture and Food Sciences, The University of Queensland, Brisbane, QLD, Australia; ^2^Plant Cell Biotechnology Department, CSIR-Central Food Technological Research Institute, Mysore, India; ^3^Algae Laboratory, Botany Department, Faculty of Science, Mansoura University, Mansoura, Egypt

**Keywords:** *Nannochloropsis*, flocculation, mushroom, chitosan, harvesting, vegan, omega-3

## Abstract

For efficient downstream processing, harvesting remains as one of the challenges in producing *Nannochloropsis* biomass, a microalga with high-value omega-3 oils. Flocculation is an effective, low-energy, low-cost method to harvest microalgae. Chitosan has been shown to be an effective food-grade flocculant; however, commercial chitosan is sourced from crustaceans, which has disadvantages including concerns over heavy-metal contamination. Thus, this study tests the flocculation potential of mushroom chitosan. Our results indicate a 13% yield of chitosan from mushroom. The identity of the prepared chitosan was confirmed by Fourier-transform infrared (FTIR) spectroscopy. Furthermore, results show that mushroom chitosan can be an alternative flocculant with >95% flocculation efficiency when tested in 100-mL jar and 200-L vertical column photobioreactor. Applications in a 2000-L raceway pond demonstrated that thorough mixing of mushroom chitosan with the algal culture is required to achieve efficient flocculation. With proper mixing, mushroom chitosan can be used to produce food-grade *Nannochloropsis* biomass suitable for the production of vegan omega-3 oils as a fish oil alternative.

## Introduction

Microalgae are photosynthetic microorganisms that grow in various environments. In recent years, research on microalgae has shifted from their use as biofuel to the production of nutraceuticals such as omega-3 fatty acids, carotenoids, and protein. *Nannochloropsis* sp. is a marine microalga, which contains high amounts of omega-3 fatty acids in the form of eicosapentaenoic acid (EPA) that has been proposed as a suitable vegan alternative for fish oil (Chua and Schenk, 2017). Its protein content can reach 36% of the biomass (Schulze et al., 2016) and even 46% according to our own data. Because of these abundant high-value products, *Nannochloropsis* sp. has gained interest from investors for large-scale cultivation.

In large-scale microalgae cultivation systems, one of the main challenges is harvesting the cells (Mathimani and Mallick, 2018). Conventional methods of harvesting include filtration and centrifugation. However, because of the small cell size (2–5 μm) of *Nannochloropsis*, these conventional harvesting methods require expensive equipment, which are also energy-intensive. Hence, harvesting can cost up to 30% of the total capital investment (Milledge and Heaven, 2013). An easy, simple, and low-cost method of harvesting is by flocculation (Vandamme et al., 2013). The most common and cheapest flocculant used is alum (Vandamme et al., 2013). However, this method contaminates the final harvested biomass with high amounts of aluminum, which makes the product not suitable for human and animal consumption. Another well-studied flocculant is chitosan (Vandamme et al., 2013; Chua et al., 2019). Chitosan is a linear polysaccharide derived from the deacetylation of the abundant natural polymer chitin, which is mainly composed of *N*-acetyl-D-glucosamine monomer units (Dimzon and Knepper, 2015). Numerous research papers have already proven the effectivity of chitosan to flocculate microalgae cells (Şirin et al., 2012; Xu et al., 2013). We have previously shown the importance of the pH of the chitosan–microalgae mixture to have high flocculation efficiencies (Chua et al., 2019). All chitosan samples tested were sourced from crustacean shells. However, crustacean-sourced chitosan has several disadvantages including heavy metal contamination (Ghormade et al., 2017). Previous studies have shown that chitosan can also be extracted from mushrooms (Yen and Mau, 2006; Erdogan et al., 2017) or even mushroom wastes (Wu et al., 2004). Thus, in this study, we produced and tested the effectiveness of mushroom chitosan for flocculating *Nannochloropsis* cells. The results were further verified in large-scale cultures, i.e., a 200-L vertical column photobioreactor culture and a 2000-L raceway pond culture.

## Materials and Methods

### Microalgae Culture

*Nannochloropsis oceanica* BR2 (Genbank accession JQ423160) was obtained from the microalgae culture collection of the University of Queensland Algae Biotechnology culture collection (Lim et al., 2012; Brown et al., 2019). The species was initially grown in a 250-mL flask using 20 g/L Ocean Nature Sea Salt (Aquasonic Pty. Ltd., NSW, Australia) enriched with f/2 medium (per L water): 75 mg NaNO_3_, 5 mg NaH_2_PO_4_⋅H_2_O, 30 mg Na_2_SiO_3_⋅9H_2_O, 3.15 mg FeCl_3_⋅6H_2_O, 4.36 mg Na_2_EDTA⋅2H_2_O, 9.8 μg CuSO_4_⋅5H_2_O, 6.3 μg Na_2_MoO_4_⋅2H_2_O, 22 μg ZnSO_4_⋅7H_2_O, 10 μg CoCl_2_⋅6H_2_O, 180 μg MnCl_2_⋅4H_2_O, 200 mg thiamine HCl, 1 μg biotin, and 1 μg cyanocobalamin (Guillard, 1975; Ma et al., 2018; Chua et al., 2019). The culture was continuously illuminated with fluorescent light (70 μmol photons m^–2^s^–1^) and aerated with filtered (through 0.2-μm pore size membrane filter) air. The culture was then gradually scaled up to the larger volumes (2 and 20 L) until it was transferred to outdoor cultures with volumes of 200 and 2000 L. The 200-L culture was grown in a vertical column photobioreactor with a diameter of 36 cm and a height of 2 m. The culture was maintained at pH 8.2 for optimum growth by bubbling CO_2_ (food-grade, 99.99% pure) at 1 vvm. Apart from maintaining the pH, CO_2_ was also the sole source of carbon for the culture. On the other hand, the 2000-L culture was grown in a 10 m^2^ raceway pond that had 1 m wide channels and a depth of 10 cm. The pond was mixed using an air-lift system and pH was controlled and maintained at 8 by automatic additions of CO_2_.

### Preparation of Chitosan From Mushroom

Chitosan was prepared from 50 g of Shiitake mushroom powder (Austral Herbs, NSW, Australia) following the method by Mohammed et al. (2013) with some modifications. Briefly, the mushroom powder was mixed with 5% NaOH solution in 1:8 ratio of powder to NaOH solution. The mixture was stirred at 120 r/min for 2 h at 60°C. Then, the sample was washed three times with distilled water. The crude chitin was deacetylated by refluxing in 50% (w/v) NaOH for 2 h at 100°C. The resulting liquor was then centrifuged, and the pellet was continuously washed until the pH was neutral. Finally, the pellet was lyophilized to obtain the crude chitosan. The entire procedure was carried out with 500 g mushroom powder for testing in the large-scale microalgal cultures.

### Characterization of the Prepared Mushroom Chitosan

The crude mushroom chitosan was characterized using a Fourier-transform infrared (FTIR) spectrophotometer (Thermo Scientific Nicolet 700) fitted with an attenuated total reflectance accessory and a diamond crystal internal reflection element. The resulting spectrum was compared to the commercial chitosan (Sigma).

### Elemental Analyses of Chitosan

Elemental analyses for heavy metals in chitosan samples were performed in duplicates as previously described (Aslam et al., 2019). Included in the analyses were two crustacean chitosan samples (Sample 1: Biomedical Chitosan, Australia; Sample 2: Qingdao Yunzhou Biochemistry Co., Ltd., China) and the mushroom-derived chitosan from the present study (Sample 3).

### Testing the Prepared Mushroom Chitosan for *Nannochloropsis* Flocculation

A similar method was used to test for the flocculation efficiency of the prepared mushroom chitosan as described in Chua et al. (2019). The optimized parameters (chitosan concentration of 25 ppm, culture optical density of 2, adjustment of pH to 6 after chitosan addition, and increase of final pH to 10 after mixing the chitosan) were used for the test.

The prepared mushroom chitosan was compared to commercial chitosan and mushroom powder. All samples were suspended in 1% acetic acid. Samples were collected at mid-height at 5, 15, and 30 min and the absorbance of the samples was measured at 440 nm to evaluate the flocculation efficiency. The flocculation efficiency was calculated using Eq. 1:

(1) $$\mathrm{Flocculation}\,\mathrm{efficiency}\,\left(\text{in}\%\right)=\left(1-\frac{O\,D_{t}}{O\,D_{0}}\right)\times 100$$

where *OD*_0_ and *OD*_t_ are the OD values of the cultures before and after the flocculation test, respectively. The culture absorbance was measured at 440 nm since this is the absorption maximum of chlorophyll *a* which is abundantly present in *Nannochloropsis*.

Further, a pilot scale testing of mushroom chitosan for flocculation was performed on *Nannochloropsis* cultivated in a 200 L vertical column photo-bioreactor and 2000 L open raceway pond. Both these cultures were maintained at pH 8 through CO_2_ supplementation. The final pH of the cultures was set by adding KOH. The same method was used to calculate the flocculation efficiency.

### Fatty Acid Quantification and Profiling

Fatty acid methyl esters from chitosan-harvested *N. oceanica* BR2 biomass were quantified by gas chromatography-mass spectrometry (GC-MS) as previously described (Ma et al., 2018). The analysis was done in triplicates.

### Statistical Analysis

The lab scale flocculation test was performed in triplicates. Tukey’s multiple comparisons test was used to test the significance among groups. Comparisons with *p*-values < 0.05 considered as statistically significant.

## Results

### Preparation of Mushroom Chitosan

**FIGURE 1:**
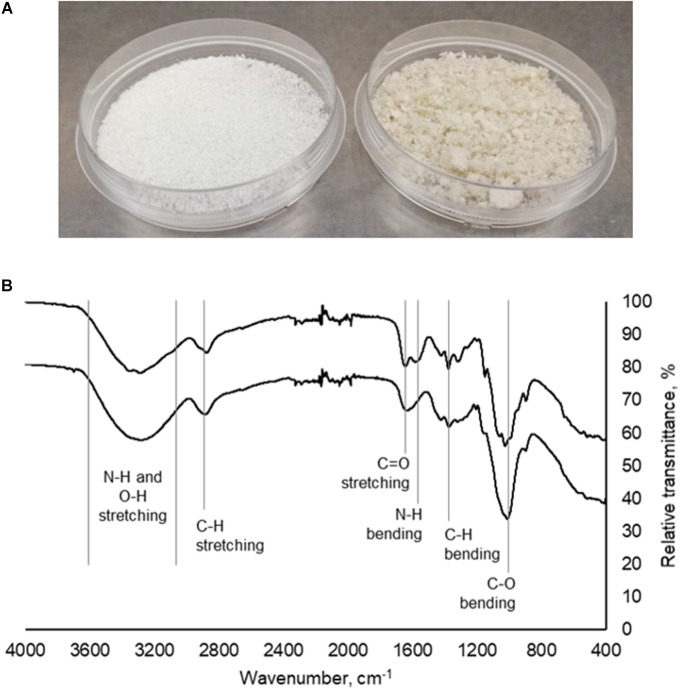
**(A)** Commercial chitosan (left) and crude mushroom chitosan (right). **(B)** Infrared spectra of commercial chitosan (top) and mushroom chitosan (bottom).

Mushroom chitosan was prepared from mushroom chitin via alkaline treatment. From the mushroom powder used, 17.22 g of crude extract was obtained after the first alkaline treatment. The first alkaline treatment was necessary to remove the protein contaminants (Wu et al., 2004; Mohammed et al., 2013; Erdogan et al., 2017). The amount of crude extract was further reduced after the second alkaline treatment to 6.16 g, which equates to a 12.32% yield. For the 500 g-mushroom powder, 157.02 g of crude chitin were obtained resulting in a 31.4% yield. After the second alkaline treatment, 68 g of crude chitosan were obtained for a final yield of 13.60%. Figure 1A shows the appearance of the mushroom chitosan after it has been lyophilized. The final yields obtained for both the small scale and large-scale extractions were comparable to those in literature (Yen and Mau, 2006; Di Mario et al., 2008).

Infrared spectroscopy (Figure 1B) results indicated that chitosan was successfully prepared from the extracted mushroom chitin. Peaks at 3400–3200 cm^–1^ correspond to the N–H and O–H stretching. The peaks around the 1660 cm^–1^ region correspond to the C = O stretching from the amide group while the peak at 1600 cm^–1^ is the amine peak (Dimzon and Knepper, 2015). Finally, the peaks at 1024, 1373, and 2870 cm^–1^ correspond to the C–O bending, C–H bending, and C–H stretching from the polymer backbone, respectively.

### Flocculation Efficiency of the Prepared Crude Mushroom Chitosan

**FIGURE 2:**
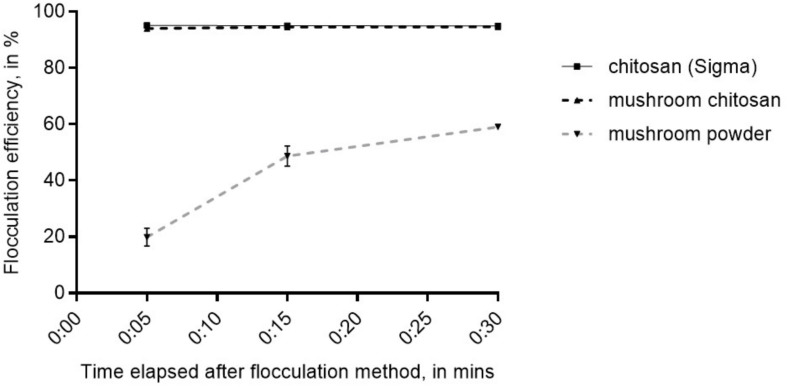
Flocculation efficiency for mushroom chitosan compared to the commercial chitosan from crustacean. Shown are mean values ± SE of three replicates.

The crude mushroom chitosan was tested on *N. oceanica* BR2 using previously optimized conditions which were: culture OD of 2, 25 ppm chitosan, adjusting the pH to 6 after chitosan addition, and increasing the final pH to 10 after mixing the chitosan into the culture (Chua et al., 2019). Similar to the results in Chua et al. (2019), no flocculation was observed without increasing the final pH to 10. Results indicated that that the crude mushroom chitosan can induce flocculation similar to the commercial chitosan (*p* > 0.05) with flocculation efficiency values > 94% after 5 min (Figure 2). On the other hand, the mushroom powder only yielded an average flocculation efficiency value of 66% even after 30 min.

### Testing of Mushroom Chitosan in a Pilot-Scale Harvesting

**FIGURE 3:**
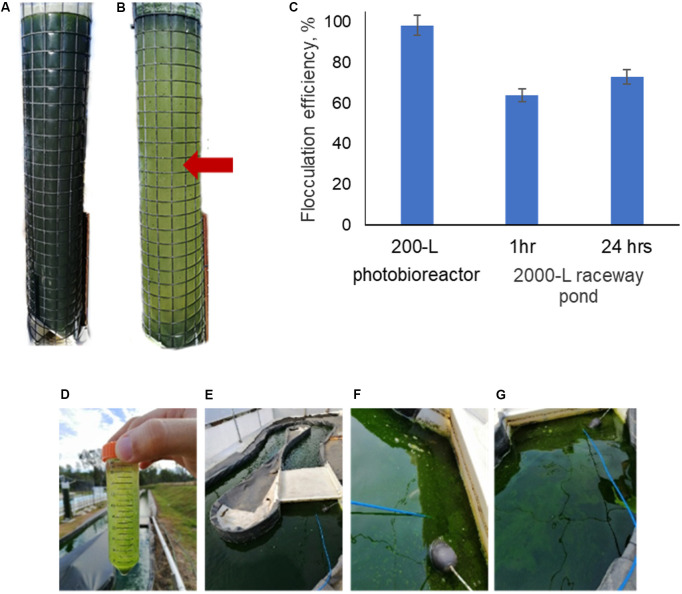
Mushroom chitosan-mediated flocculation of *N. oceanica* in a 200-L tower culture: **(A)** before flocculation and **(B)** after flocculation. The resulting flocculation efficiencies are presented in **(C)**. The red arrow indicates the sampling point; and in a 2000-L raceway pond: **(D)** testing on a 50-mL sample, **(E)** before flocculation, **(F,G)** after flocculation.

The effectiveness of mushroom chitosan to induce flocculation of *N. oceanica* BR2 was tested at pilot scale using a 200-L vertical column photo-bioreactor and a 2000-L open raceway pond. Figures 3A,B show the 200-L column photo-bioreactor before and after the flocculation procedure, respectively. In this case, a flocculation efficiency of 98.3% was achieved. As for the 2000-L raceway pond, it had an initial OD of 2.9. So, a 50-mL sample was obtained before testing. Flocculation was observed after adding chitosan into the 50-mL sample as shown in Figure 3D indicating that 25 ppm of mushroom chitosan is still effective. Mixing was performed by using an air-lift system, and the chitosan was poured in and mixed for 10 min. Figures 3F,G show some of the flocs that formed after the chitosan was added and these flocs were not present before chitosan addition as shown in Figure 3E. The entire procedure yielded 64% flocculation efficiency after 1 h of settling. After 24 h, samples were collected at different points of the pond, and the average OD was 0.787, yielding 73% flocculation efficiency. Figure 3C summarizes the flocculation results of the 200- and 2000-L culture.

To determine which type of chitosan (mushroom- or crustaceae-derived) is a safer option for human consumption, elemental analyses were performed using two samples of crustacean and one sample of mushroom chitosan from the present study. These showed that heavy metals varied greatly for the two crustacean-derived samples, with chromium and nickel levels as high as 47.44 and 27.21 mg/kg, respectively, while mushroom-derived chitosan did not contain any concerning heavy metal contamination (Supplementary Table S1).

### Fatty Acid Profiling of Mushroom Chitosan-Harvested *N. oceanica* BR2 Biomass

The mushroom chitosan-harvested biomass has EPA levels up to 41.3(±0.3)% of the total fatty acid content. Other fatty acids detected were palmitic acid (C16:0, 15.0 ± 0.6%), palmitoleic acid (C16:1, 34.4 ± 0.8%), oleic acid (C18:1, 2.8 ± 0.3%), linoleic acid (C18:2, 1.8 ± 0.2%), and arachidonic acid (C20:4n-6, 4.7 ± 0.3%). Supplementary Table S2 lists the fatty acid profile of the mushroom-harvested *N. oceanica* BR2 biomass together with profiles from other studies for comparison.

## Discussion

Chitin is popularly known to be extracted from the shells of crustaceans such as crabs and shrimps. Earlier estimates have shown that more than 80,000 tons of chitin is obtained from marine by-products (Ghormade et al., 2017). However, there are some disadvantages to marine-derived chitosan including seasonal variation and possible heavy metal contamination (Ghormade et al., 2017; Abo Elsoud and El Kady, 2019). This was also confirmed in the present study (Supplementary Table S1). In addition, fungal chitosan is suitable for vegans and is free from allergenic shrimp protein, which can be included in the final harvested biomass (Arcidiacono and Kaplan, 1992; Dhillon et al., 2013).

To theoretically estimate the comparative production expenses of chitosan from mushroom and crustaceans, the literature outlines that due to the inconsistent structure of chitin and chitosan from crustaceans, fungal (mushroom in this case) may represent a better alternative (Di Mario et al., 2008; Hassainia et al., 2018; Jones et al., 2020). Furthermore, fluctuations in seasonal supply of various animal sources and challenges in raw material standardization cause high variability in terms of deacetylation degree and molecular mass. These results may interfere in final flocculation efficiency of the chitosan. Unlike crustacean chitin, fungal chitin has more consistent physical and chemical properties, is not limited by seasonal and regional variation, and does not require the aggressive acid treatment that crustacean chitin needs for purification and demineralization to remove calcium carbonate and other minerals (Di Mario et al., 2008; Hassainia et al., 2018; Jones et al., 2020). In addition, from an environmental economics and sustainability point of view, crustacean chitosan production is likely to generate more waste than fungal chitosan. In the traditional process of chitin extraction from crustaceans, calcium and proteins are removed by HCl and NaOH, respectively. The remaining material is usually bleached with KMnO_4_ or H_2_O_2_ and deacetylation is performed with hot concentrated alkaline or acidic solution. These harsh treatments can result in considerable amounts of wastes and deleterious trace contaminants (Bierhalz et al., 2016). Therefore, chitosan produced from mushroom waste is safer, more environmentally friendly, more reliable in its supply, and suitable for vegetarians.

A difference in color between the crude mushroom chitosan and the commercial chitosan was observed, which could be because the mushroom chitosan was not purified. The yellowish color was also obtained by Yen and Mau (2006). Further purification may be conducted by refluxing the crude powder in HCl or acetic acid (Wu et al., 2004; Darwesh et al., 2018). Decolorization may also be performed to improve the color (Yen and Mau, 2006; Mohammed et al., 2013). However, these processes will increase the cost of producing the chitosan flocculant. Nevertheless, even without the purification step, IR spectroscopy revealed the successful preparation of mushroom chitosan. The same characteristic peaks were observed in the IR spectrum of the mushroom chitosan when compared to the commercial chitosan.

Chitosan has been demonstrated as a good alternative bio-flocculant (Xu et al., 2013; Chua et al., 2019). Our flocculation test results confirm that mushroom chitosan was successfully prepared and has similar flocculation properties as the commercial chitosan. Mushroom powder did not flocculate the cells, which indicates that chitosan is the active ingredient and can only be obtained after deacetylation. Recently, Pugazhendhi et al. (2019) discussed the chemical mechanism of cationic polymers for microalgae flocculation. Because chitosan is cationic at acidic pH; thus, the lowering of the pH was necessary. In another study by Blockx et al. (2018), they demonstrated that high pH is necessary to flocculate microalgae in seawater medium. Thus, it was necessary to increase the pH after chitosan addition. This demonstrates that mushroom chitosan can indeed be used to harvest *Nannochloropsis* biomass that would then be suitable for vegetarians or even vegans. The fatty acid profile, which was not different from those in literature, further supports that the mushroom chitosan-harvested biomass can indeed serve as an alternative source of fish oil.

Previous studies (Table 1) have tested different flocculants to harvest *Nannochloropsis* spp. In the current study, mushroom chitosan has resulted in almost similar flocculation efficiency of >94% as compared to the previously reported chemical-based flocculants such as Al_2_(SO_4_)_3_ and FeCl_3_, but at much lower concentrations. Therefore, in this case, chitosan has advantage over chemical flocculants, which are not recommended for food-grade applications of harvested biomass. On the other hand, mung bean protein concentrate can be avoided since it can drive the food debate over its use for harvesting purpose. Tanfloc showed a comparable flocculation efficiency and at an even lower concentration compared to the mushroom chitosan concentration used in this study. However, it is not clear if Tanfloc can be used in food production as it is currently sold for water and wastewater treatment (TANAC, S.A.^1^). To our knowledge, this is the first study to report the use of mushroom chitosan for microalgae flocculation.

**TABLE 1:** Comparison of different flocculants used for *Nannochloropsis* sp. harvesting.

**Flocculant**	**Concentration**	**Flocculation efficiency**	**References**
Aluminum sulfate	82.5 ppm	>95%	Chua et al., 2019
Ferric chloride	82.5 ppm	>95%	Chua et al., 2019
Tanfloc	10 ppm	98%	Roselet et al., 2016
AFlok-BP1	160 ppm	92%	Fuad et al., 2018
Mung bean protein concentrate	20 mL/L	>90%	Kandasamy and Shaleh, 2017
γ-Polyglutamic acid	22 ppm	96%	Zheng et al., 2012
Mushroom chitosan	25 ppm	>94%	This study

Higher flocculation efficiencies were observed in the 200-L vertical column photobioreactor compared to the air-lift raceway pond. The low flocculation efficiency was likely caused by the suboptimal mixing of the chitosan into the pond culture, as the mixing was significantly more efficient in the 50-mL and 200-L cultures (>95 and 98.3% yield, respectively). The raceway pond was slowly mixed using an air-lift system achieved with microbubbles, which may have also affected the performance since the bubbles disrupted the large flocs. Even after 24 h, the flocculation efficiency did not reach >90%. This result clearly indicated that the cells have not interacted properly with the chitosan. Bleeke et al. (2015) and Pugazhendhi et al. (2019) discussed the importance of mixing speed, intensity, and time. Mixing using a paddle-wheel system may provide a better flocculation performance as the mechanism less disruptive to the large flocs as they pass through. Koley et al. (2017) have demonstrated the effectivity of chitosan with flocculation efficiencies of ∼90% to flocculate *Scenedesmus obliquus* and *Chlorella vulgaris* cultured in raceway ponds. However, the cultures had to be pumped into 1000-L tanks, which was easier to mix with a large motor-driven stirrer. Further optimization on the mixing of the chitosan into the pond culture would be necessary along with economic feasibility studies to improve the attractiveness of chitosan for use in microalgae harvesting.

## Conclusion

Mushroom chitosan was prepared by extracting and deacetylating chitin from mushroom powder and was verified using FTIR. Results showed that the prepared mushroom chitosan had similar flocculation efficiency as commercial crustacean-derived chitosan. Furthermore, chitosan can be sustainably prepared utilizing the wastes from mushroom industries and using it for harvesting promotes the chemical-free harvesting protocol for microalgae for food and/or feed applications. While chitosan was also found suitable for harvesting of large culture volumes, the requirement for efficient mixing should be considered. The availability of mushroom chitosan harvested *Nannochloropsis* offer an affordable and sustainable fish oil replacement product suitable for vegans.

## Data Availability Statement

The original contributions presented in the study are included in the article/Supplementary Material. Further inquiries can be directed to the corresponding author.

## Author Contributions

EC conducted the experiments, analyzed the results, and wrote the original draft. AS assisted in the large-scale experiments. EE assisted in preparing the mushroom chitosan. ST-H prepared the resources for the large-scale experiments. PS supervised the entire project and acquired the necessary funding. All authors reviewed the manuscript.

## Conflict of Interest

The authors declare that the research was conducted in the absence of any commercial or financial relationships that could be construed as a potential conflict of interest.

## Footnotes

1 http://www.tanac.com.br/en/

## References

[B1] Abo Elsoud M. M. El Kady E. M. (2019). Current trends in fungal biosynthesis of chitin and chitosan. *Bull. Natl. Res. Cent.* 43:59 10.1186/s42269-019-0105-y

[B2] Arcidiacono S. Kaplan D. L. (1992). Molecular weight distribution of chitosan isolated from *Mucor rouxii* under different culture and processing conditions. *Biotechnol. Bioeng.* 39 281–286. 10.1002/bit.260390305 18600943

[B3] Aslam A. Thomas-Hall S. R. Mughal T. Zaman Q. U. Ehsan N. Javied S. (2019). Heavy metal bioremediation of coal-fired flue gas using microalgae under different CO_2_ concentrations. *J. Environ. Manag.* 241 243–250. 10.1016/j.jenvman.2019.03.118 31005725

[B4] Bierhalz A. C. K. Westin C. B. Moraes A. M. (2016). Comparison of the properties of membranes produced with alginate and chitosan from mushroom and from shrimp. *Int. J. Biol. Macromol.* 91 496–504. 10.1016/j.ijbiomac.2016.05.095 27240752

[B5] Bleeke F. Milas M. Winckelmann D. Klöck G. (2015). Optimization of freshwater microalgal biomass harvest using polymeric flocculants. *Int. Aquat. Res.* 7 235–244. 10.1007/s40071-015-0108-8

[B6] Blockx J. Verfaillie A. Thielemans W. Muylaert K. (2018). Unravelling the mechanism of chitosan-driven flocculation of microalgae in seawater as a function of pH. *ACS Sustainab. Chem. Eng.* 6 11273–11279. 10.1021/acssuschemeng.7b04802

[B7] Brown R. B. Wass T. J. Thomas-Hall S. R. Schenk P. M. (2019). Chromosome-scale genome assembly of two Australian *Nannochloropsis oceanica* isolates exhibiting superior lipid characteristics. *Microbiol. Resour. Announc.* 8:48. 10.1128/MRA.01288-19 31776227 PMC6883114

[B8] Chua E. T. Eltanahy E. Jung H. Uy M. Thomas-Hall S. R. Schenk P. M. (2019). Efficient harvesting of *Nannochloropsis* microalgae via optimized chitosan-mediated flocculation. *Glob. Challeng.* 3:1800038. 10.1002/gch2.201800038 31565353 PMC6383959

[B9] Chua E. T. Schenk P. M. (2017). A biorefinery for *Nannochloropsis*: induction, harvesting, and extraction of EPA-rich oil and high-value protein. *Bioresour. Technol.* 244 1416–1424. 10.1016/j.biortech.2017.05.124 28624245

[B10] Darwesh O. M. Sultan Y. Y. Seif M. M. Marrez D. A. (2018). Bio-evaluation of crustacean and fungal nano-chitosan for applying as food ingredient. *Toxicol. Rep.* 5 348–356. 10.1016/j.toxrep.2018.03.002 29854604 PMC5977412

[B11] Dhillon G. S. Kaur S. Brar S. K. Verma M. (2013). Green synthesis approach: extraction of chitosan from fungus mycelia. *Crit. Rev. Biotechnol.* 33 379–403. 10.3109/07388551.2012.717217 23078670

[B12] Di Mario F. Rapanà P. Tomati U. Galli E. (2008). Chitin and chitosan from Basidiomycetes. *Int. J. Biol. Macromol.* 43 8–12. 10.1016/j.ijbiomac.2007.10.005 18023863

[B13] Dimzon I. K. D. Knepper T. P. (2015). Degree of deacetylation of chitosan by infrared spectroscopy and partial least squares. *Int. J. Biol. Macromol.* 72 939–945. 10.1016/j.ijbiomac.2014.09.050 25316417

[B14] Erdogan S. Kaya M. Akata I. (2017). Chitin extraction and chitosan production from cell wall of two mushroom species (*Lactarius vellereus* and *Phyllophora ribis*). *AIP Conf. Proc.* 1809:020012 10.1063/1.4975427

[B15] Fuad N. Omar R. Kamarudin S. Harun R. Idris A. Wan Azlina W. A. K. G. (2018). Effective use of tannin based natural biopolymer, AFlok-BP1 to harvest marine microalgae *Nannochloropsis* sp. *J. Environ. Chem. Eng.* 6 4318–4328. 10.1016/j.jece.2018.06.041

[B16] Ghormade V. Pathan E. K. Deshpande M. V. (2017). Can fungi compete with marine sources for chitosan production? *Int. J. Biol. Macromol.* 104 1415–1421. 10.1016/j.ijbiomac.2017.01.112 28143744

[B17] Guillard R. R. L. (1975). *Culture of Phytoplankton For Feeding Marine Invertebrates, In Culture Of Marine Invertebrate Animals.* New York, NY: Plenum Press.

[B18] Hassainia A. Satha H. Boufi S. (2018). Chitin from agaricus bisporus: extraction and characterization. *Int. J. Biol. Macromol.* 117 1334–1342. 10.1016/j.ijniomax.2017.11.172 29197571

[B19] Hulatt C. J. Wijffels R. H. Bolla S. Kiron V. (2017). Production of fatty acids and protein by *Nannochloropsis* in flat-plate photobioreactors. *PLoS One* 12:e0170440. 10.1371/journal.pone.0170440 28103296 PMC5245880

[B20] Jones M. Kujundzic M. John S. Bismarck A. (2020). Crab vs. mushroom: a review of crustacean and fungal chitin in wound treatment. *Mar. Drugs* 18:64. 10.3390/md18010064 31963764 PMC7024172

[B21] Kandasamy G. Shaleh S. R. M. (2017). Harvesting of the microalga *Nannochloropsis* sp. by bioflocculation with mung bean protein extract. *Appl. Biochem. Biotech.* 182 587–597. 10.1007/s12010-016-2346-7 27957653

[B22] Koley S. Prasad S. Bagchi S. K. Mallick N. (2017). Development of a harvesting technique for large-scale microalgal harvesting for biodiesel production. *RSC Adv.* 7 7227–7237. 10.1039/C6RA27286J

[B23] Lim D. K. Y. Garg S. Timmins M. Zhang E. S. B. Thomas-Hall S. R. Schuhmann H. (2012). Isolation and evaluation of oil-producing microalgae from subtropical coastal and brackish waters. *PLoS One* 7:e40751. 10.1371/journal.pone.0040751 22792403 PMC3394722

[B24] Ma R. Thomas-Hall S. R. Chua E. T. Eltanahy E. Netzel M. E. Netzel G. (2018). LED power efficiency of biomass, fatty acid, and carotenoid production in *Nannochloropsis* microalgae. *Bioresour. Technol.* 252, 118–126. 10.1016/j.biortech.2017.12.096 29306714

[B25] Mathimani T. Mallick N. (2018). A comprehensive review on harvesting of microalgae for biodiesel - key challenges and future directions. *Renew. Sustain. Energy Rev* 91 1103–1120. 10.1016/j.rser.2018.04.083

[B26] Milledge J. J. Heaven S. (2013). A review of the harvesting of micro-algae for biofuel production. *Rev. Environ. Sci. Biotechnol.* 12 165–178. 10.1007/s11157-012-9301-z

[B27] Mohammed M. H. Williams P. A. Tverezovskaya O. (2013). Extraction of chitin from prawn shells and conversion to low molecular mass chitosan. *Food Hydrocoll.* 31 166–171. 10.1016/j.foodhyd.2012.10.021

[B28] Pugazhendhi A. Shobana S. Bakonyi P. Nemestothy N. Xia A. Banu J. (2019). A review on chemical mechanism of microalgae flocculation via polymers. *Biotechnol. Rep.* 21:e00302. 10.1016/j.btre.2018.e00302 30671358 PMC6328355

[B29] Roselet F. Burkert J. Abreu P. C. (2016). Flocculation of *Nannochloropsis oculata* using a tannin-based polymer: bench scale optimisation and pilot scale reproducibility. *Biomass Bioenergy* 87 55–60. 10.1016/j.biombioe.2016.02.015

[B30] Schulze P. S. C. Pereira H. G. C. Santos T. F. C. Schueler L. Guerra R. Barreira L. A. (2016). Effect of light quality supplied by light emitting diodes (LEDs) on growth and biochemical profiles of *Nannochloropsis oculata* and *Tetraselmis chuii*. *Algal Res.* 16 387–398. 10.1016/j.algal.2016.03.034

[B31] Shen P.-L. Wang H.-T. Pan Y.-F. Meng Y.-Y. Wu P.-C. Xue S. (2016). Identification of characteristic fatty acids to quantify triacylglycerols in microalgae. *Front. Plant Sci.* 7:162. 10.3389/fpls.2016.00162 26941747 PMC4761805

[B32] Şirin S. Trobajo R. Ibanez C. Salvadó J. (2012). Harvesting the microalgae *Phaeodactylum tricornutum* with polyaluminum chloride, aluminium sulphate, chitosan and alkalinity-induced flocculation. *J. Appl. Phycol.* 24 1067–1080. 10.1007/s10811-011-9736-6

[B33] Vandamme D. Foubert I. Muylaert K. (2013). Flocculation as a low-cost method for harvesting microalgae for bulk biomass production. *Trends Biotechnol.* 31 233–239. 10.1016/j.tibtech.2012.12.005 23336995

[B34] Wu T. Zivanovic S. Draughon F. A. Sams C. E. (2004). Chitin and chitosan value-added products from mushroom waste. *J. Agric. Food Chem.* 52 7905–7910. 10.1021/jf0492565 15612774

[B35] Xu Y. Purton S. Baganz F. (2013). Chitosan flocculation to aid the harvesting of the microalga *Chlorella sorokiniana*. *Bioresour. Technol.* 129 296–301. 10.1016/j.biortech.2012.11.068 23262003

[B36] Yen M.-T. Mau J.-L. (2006). Preparation of fungal chitin and chitosan from shiitake stipes. *Fung. Sci.* 21 1–11. 10.7099/FS.200612.0001

[B37] Zheng H. Gao Z. Yin J. Tang X. Ji X. Huang H. (2012). Harvesting of microalgae by flocculation with poly (γ-glutamic acid). *Bioresour. Technol.* 112 212–220. 10.1016/j.biortech.2012.02.086 22425514

