# Supplementary material for: Effective Harvesting of Nannochloropsis Microalgae Using Mushroom Chitosan: A Pilot-Scale Study
Source: Front Bioeng Biotechnol. 2020 Jul 14;8:771. doi: 10.3389/fbioe.2020.00771 (PMC7381157; doi:10.3389/fbioe.2020.00771)
Supplement: Supplementary file 1 [file Table_1.DOCX]

**Supplementary Table S1.** Fatty acid profile of mushroom-derived chitosan-harvested *N. oceanica* BR2 biomass compared to profiles in literature.

| Fatty acid | This study | Hulatt et al. (2017) | Shen et al. (2016) |
| --- | --- | --- | --- |
| C16:0 | 15.0 (± 0.6) | 19.4( ± 0.3) | 21.6 ( ± 0.0) |
| C16:1 | 34.4 (± 0.8) | 21.8( ± 1.2) | 24 ( ± 0.0) |
| C18:1n-9 | 2.8 (± 0.3) | 0.9( ± 1.5) | 1.8 ( ± 0.0) |
| C18:2n-6 | 1.8 (± 0.2) | 2.5( ± 1.2) | 2 ( ± 0.2) |
| C20:4n-6 | 4.7 (± 0.3) | 0.3( ± 0.3) | 4 ( ± 0.1) |
| C20:5n-3 (EPA) | 41.3 (± 0.3) | 41.5( ± 2.7) | 30.5 ( ± 0.3) |

**Supplementary Table S2.** Heavy metal analysis (in mg/kg) of chitosan samples.

| Heavy metal | Sample 1 | Sample 2 | Sample 3 |
| --- | --- | --- | --- |
| As | 2.46 ± 0.28 | 7.76 ± 0.14 | 1.24 ± 0.32 |
| Cd | 1.43 ± 0.04 | 0.18 ± 0.00 | 0.15 ± 0.01 |
| Co | 0.09 ± 0.00 | 0.23 ± 0.01 | 0.37 ± 0.03 |
| Cr | 1.01 ± 0.05 | 47.44 ± 0.35 | 9.42 ± 0.27 |
| Cu | 0.37 ± 0.01 | 3.46 ± 0.07 | 3.26 ± 0.07 |
| Hg | 0.08 ± 0.01 | 0.00 ± 0.00 | 0.03 ± 0.01 |
| Mn | 0.15 ± 0.01 | 5.18 ± 0.19 | 30.65 ± 0.14 |
| Ni | 0.36 ± 0.01 | 27.21 ± 0.32 | 2.24 ± 0.13 |
| Pb | 1.29 ± 0.06 | 2.75 ± 0.15 | 1.32 ± 0.06 |
| Zn | 0.97 ± 0.03 | 1.33 ± 0.08 | 8.90 ± 0.11 |
